# Supplementary material for: High Galectin-7 and Low Galectin-8 Expression and the Combination of both are Negative Prognosticators for Breast Cancer Patients
Source: Cancers (Basel). 2020 Apr 12;12(4):953. doi: 10.3390/cancers12040953 (PMC7226378; doi:10.3390/cancers12040953)
Supplement: Supplementary file 1 [file cancers-12-00953-s001.docx]

Supplementary Materials

High Galectin-7 and Low Galectin-8 Expression and the Combination of both are Negative Prognosticators for Breast Cancer Patients

Anna Trebo, Nina Ditsch, Christina Kuhn, Helene Hildegard Heidegger, Christine Zeder-Goess, Thomas Kolben, Bastian Czogalla, Elisa Schmoeckel, Sven Mahner, Udo Jeschke
and Anna Hester

**Table S1.** Correlations of Gal‑7 expression with clinical and histological parameters.

|  | | **age at surgery** | **NST vs other** | **pT** | **pN** | **Grading** | **Intrinsic subtype** | **HER2** | **ER** | **PR** |
| --- | --- | --- | --- | --- | --- | --- | --- | --- | --- | --- |
| Gal-7 IRS cytoplasm | Correlation Coefficient | **-0,184^**^** | **-0,282^**^** | 0,049 | 0,077 | **0,179^*^** | **0,277^**^** | **0,396^**^** | -0,096 | **-0,142^*^** |
|  | Sig. (2-tailed) | 0,007 | 0,000 | 0,472 | 0,272 | 0,027 | 0,000 | 0,000 | 0,160 | 0,037 |
|  | N | 216 | 203 | 215 | 207 | 153 | 216 | 213 | 216 | 216 |
| Gal-7 IRS nucleus | Correlation Coefficient | -0,039 | **-0,143^*^** | -0,023 | 0,020 | -0,010 | -0,037 | 0,053 | 0,030 | 0,043 |
|  | Sig. (2-tailed) | 0,568 | 0,042 | 0,739 | 0,773 | 0,901 | 0,592 | 0,443 | 0,662 | 0,533 |
|  | N | 216 | 203 | 215 | 207 | 153 | 216 | 213 | 216 | 216 |

Gal-7 expression in the cytoplasm correlated significantly positive with the grading, the intrinsic subtype and the HER2 status, and negative with the age at surgery, the histological subtype, and the PR status. Gal‑7 expression in the nucleus correlated negative with the histological subtype.

**Table S2.** Correlations of Gal‑8 expression with clinical and histological parameters.

|  | | **age at surgery** | **NST vs other** | **pT** | **pN** | **Grading** | **Intrinsic subtype** | **HER2** | **ER** | **PR** |
| --- | --- | --- | --- | --- | --- | --- | --- | --- | --- | --- |
| Gal8 IRS cytoplasm | Correlation Coefficient | **-0,162^*^** | -0,065 | 0,019 | -0,023 | 0,031 | 0,037 | **0,197^**^** | 0,064 | -0,009 |
|  | Sig. (2-tailed) | 0,017 | 0,361 | 0,777 | 0,742 | 0,702 | 0,589 | 0,004 | 0,348 | 0,891 |
|  | N | 215 | 202 | 214 | 206 | 154 | 215 | 213 | 215 | 215 |
| Gal-8 IRS nucleus | Correlation Coefficient | -0,120 | 0,024 | 0,009 | 0,000 | **-0,174^*^** | -0,072 | 0,041 | **0,152^*^** | 0,113 |
|  | Sig. (2-tailed) | 0,079 | 0,731 | 0,897 | 0,998 | 0,031 | 0,294 | 0,553 | 0,026 | 0,098 |
|  | N | 215 | 202 | 214 | 206 | 154 | 215 | 213 | 215 | 215 |

Gal-8 expression in the cytoplasm correlated significantly positive with the HER2 status and negative with the age at surgery. Gal‑8 expression in the nucleus correlated positive with the ER status and negative with the grading.

**Table S3.** Multivariate analysis of DDFS concerning Gal‑7 expression in the cytoplasm.

|  | **B** | **SE** | **Wald** | **df** | **Sig.** | **Exp(B)** | **95,0% CI for Exp(B)** | |
| --- | --- | --- | --- | --- | --- | --- | --- | --- |
|  |  |  |  |  |  |  | **Lower** | **Upper** |
| Histological subtype (NST vs. non-NST) | 0,087 | 0,680 | 0,016 | 1 | 0,898 | 1,091 | 0,288 | 4,134 |
| Grading (G1 vs. G2-3) | 12,903 | 587,767 | 0,000 | 1 | 0,982 | 401627 | 0,000 |  |
| Tumor size (pT1 vs. pT2-4) | 0,370 | 0,484 | 0,583 | 1 | 0,445 | 1,447 | 0,560 | 3,737 |
| Nodal status (pN0 vs. pN1-3) | 1,100 | 0,556 | 3,919 | 1 | **0,048** | 3,004 | 1,011 | 8,929 |
| HER2 status (positive vs. negative) | -0,402 | 0,654 | 0,378 | 1 | 0,539 | 0,669 | 0,186 | 2,411 |
| ER (positive vs. negative) | -1,029 | 0,577 | 3,186 | 1 | 0,074 | 0,357 | 0,115 | 1,106 |
| PR (positive vs. negative) | -0,676 | 0,656 | 1,062 | 1 | 0,303 | 0,509 | 0,141 | 1,839 |
| Patient age (continuous) | 0,011 | 0,021 | 0,299 | 1 | 0,585 | 1,012 | 0,971 | 1,054 |
| Gal-7 expression in the cytoplasm (high vs. low) | 0,742 | 0,534 | 1,935 | 1 | 0,164 | 2,101 | 0,738 | 5,978 |

**Table S4.** Multivariate analysis of OS concerning Gal‑7 expression in the cytoplasm.

|  | **B** | **SE** | **Wald** | **df** | **Sig.** | **Exp(B)** | **95,0% CI for Exp(B)** | |
| --- | --- | --- | --- | --- | --- | --- | --- | --- |
|  |  |  |  |  |  |  | **Lower** | **Upper** |
| Histological subtype (NST vs. non-NST) | 0,226 | 0,455 | 0,246 | 1 | 0,620 | 1,253 | 0,514 | 3,058 |
| Grading (G1 vs. G2-3) | -0,517 | 0,590 | 0,769 | 1 | 0,380 | 0,596 | 0,188 | 1,894 |
| Tumor size (pT1 vs. pT2-4) | 0,224 | 0,389 | 0,333 | 1 | 0,564 | 1,251 | 0,584 | 2,680 |
| Nodal status (pN0 vs. pN1-3) | 0,953 | 0,407 | 5,480 | 1 | **0,019** | 2,593 | 1,168 | 5,756 |
| HER2 status (positive vs. negative) | 0,108 | 0,622 | 0,030 | 1 | 0,863 | 1,114 | 0,329 | 3,770 |
| ER (positive vs. negative) | -1,304 | 0,496 | 6,922 | 1 | **0,009** | 0,271 | 0,103 | 0,717 |
| PR (positive vs. negative) | 0,394 | 0,479 | 0,676 | 1 | 0,411 | 1,482 | 0,580 | 3,788 |
| Patient age (continuous) | 0,048 | 0,017 | 8,271 | 1 | **0,004** | 1,049 | 1,015 | 1,085 |
| Gal-7 expression in the cytoplasm (high vs. low) | 0,445 | 0,446 | 0,995 | 1 | 0,318 | 1,560 | 0,651 | 3,736 |

**Table S5.** Coordinates of ROC Curve exemplary for Gal‑7 expression in the cytoplasm.
Cut off points for Gal‑7 should be between 8 and 10, we found that 6 should be the better cutoff considering also Kruskal-Wallis tests.

| **Test Result Variable(s)** | **Positive if Greater Than or Equal To^a^** | **Sensitivity** | **1 - Specificity** |
| --- | --- | --- | --- |
| Gal‑7 expression | -1,00 | 1,000 | 1,000 |
|  | 0,50 | 0,957 | 0,969 |
|  | 1,50 | 0,957 | 0,961 |
|  | 2,50 | 0,714 | 0,775 |
|  | 3,50 | 0,657 | 0,674 |
|  | 5,00 | 0,486 | 0,488 |
|  | 7,00 | 0,186 | 0,202 |
|  | **8,50** | 0,071 | 0,070 |
|  | **10,50** | 0,043 | 0,039 |


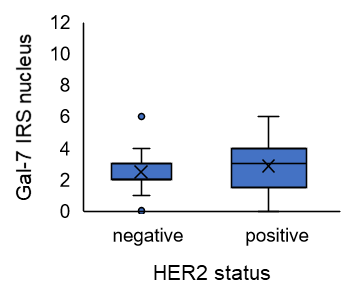


**p = 0.442**


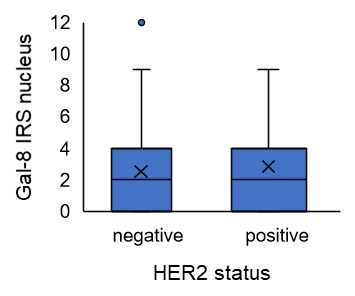


**p = 0.551**

A

B

**Figure S1**: Association of Gal 7 and Gal¬ 8 expression in the nucleus with HER2 status.
Boxplots of the mean IRS of Gal-7 (A) and Gal 8 (B) staining in the nucleus +/- SD dependent on the HER2 status of the tumor are shown.


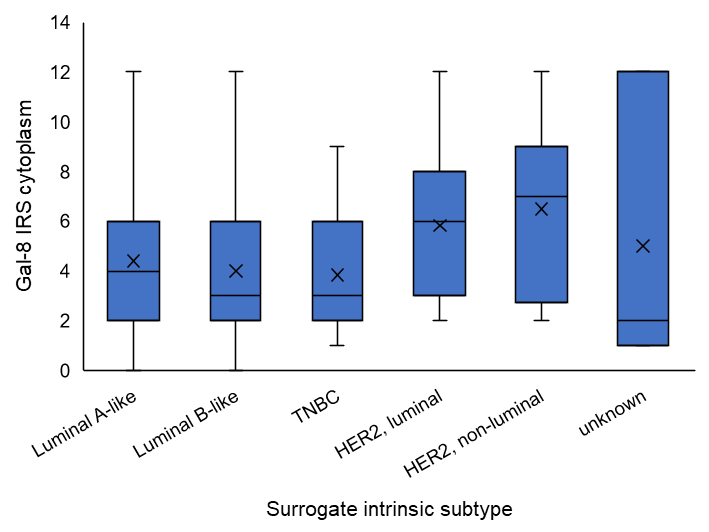


p = 0.055

**Figure S2**: Association of Gal-8 expression in the cytoplasm to the different surrogate intrinsic subtypes.
Boxplots of the mean IRS of Gal-8 staining in the cytoplasm +/- SD dependent on the surrogate intrinsic subtype of the tumor is shown. HER2-positive, both luminal and non-luminal tumors show the trend of higher Gal-8 expression in the cytoplasm compared to the other subtypes.


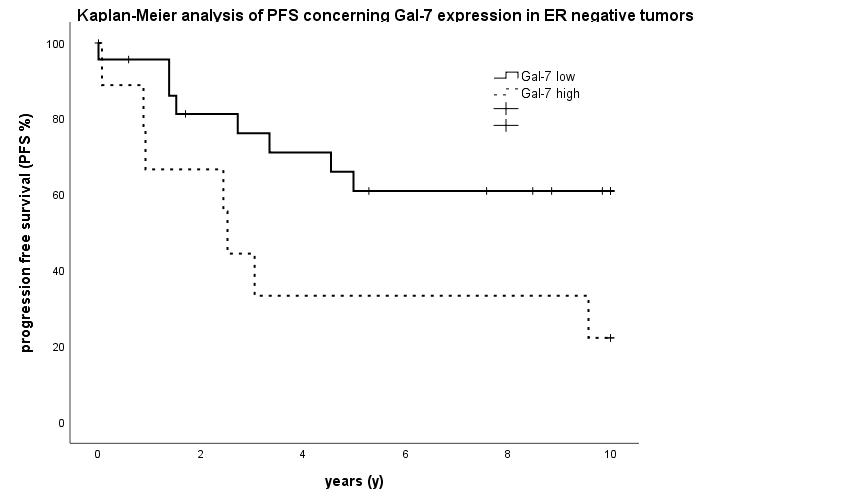


p = 0.036

**Figure S3**: PPT. Association of Gal-7 expression in the cytoplasm in ER negative tumors to the clinical outcome.
Kaplan-Meier analysis of PFS in Gal-7 high and low expressing ER negative tumors (in the cytoplasm) is shown. ER negative tumors with high Gal-7 expression in the cytoplasm showed a significantly impaired PFS.


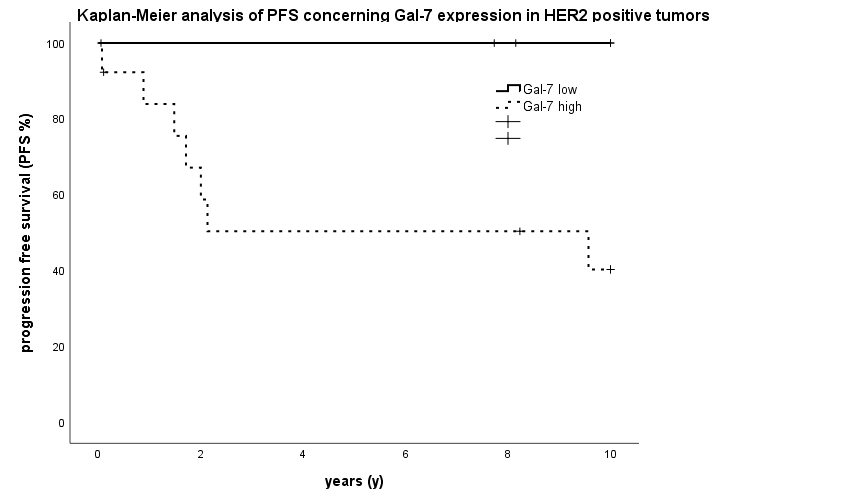


p = 0.084

**Figure S4**: Association of Gal-7 expression in the cytoplasm in HER2 positive tumors to the clinical outcome.
Kaplan-Meier analysis of PFS in Gal-7 high and low expressing HER2 positive tumors (in the cytoplasm) is shown. HER2 positive tumors with high Gal-7 expression in the cytoplasm showed the trend to an impaired PFS.


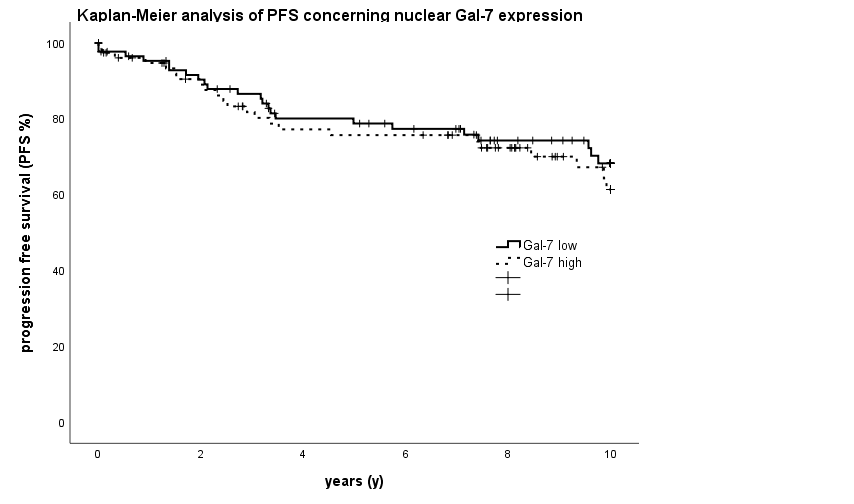


p = 0.525


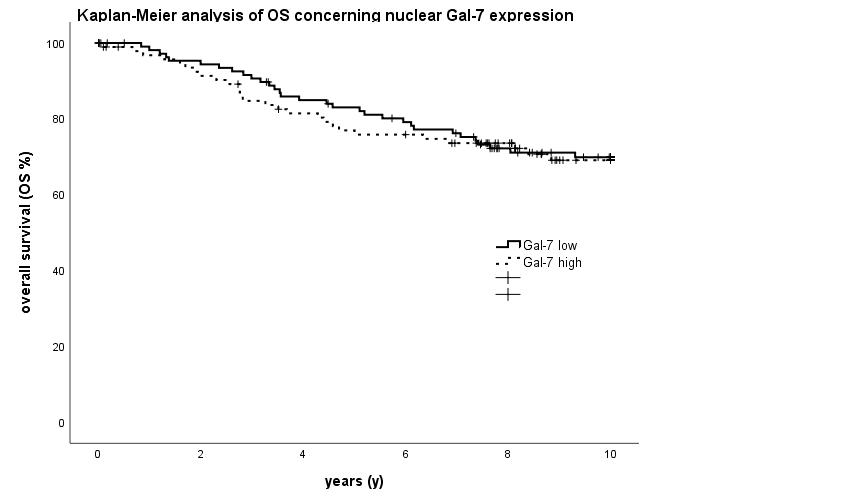


p = 0.809


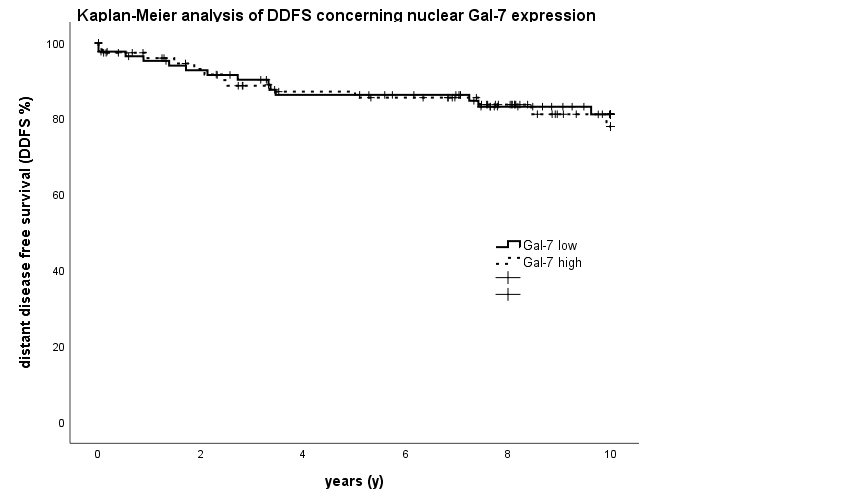


p = 0.789

**Figure S5**: Association of Gal-7 expression in the nucleus to the clinical outcome.
Kaplan-Meier analysis of OS (top left), PFS (top right) and DDFS (bottom left) in Gal-7 high and low expressing tumors (in the nucleus) is shown. No significant differences are seen.


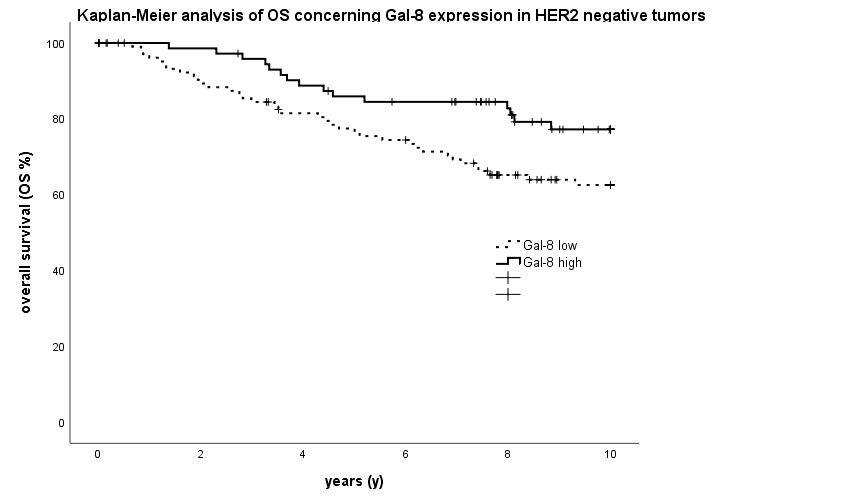


p = 0.029

**Figure S6:** Association of Gal-8 expression in the cytoplasm in HER2 negative tumors to the clinical outcome.
Kaplan-Meier analysis of OS in Gal-8 high and low expressing HER2 negative tumors (in the cytoplasm) is shown. HER2 negative tumors with high Gal-8 expression in the cytoplasm showed a significantly improved OS.


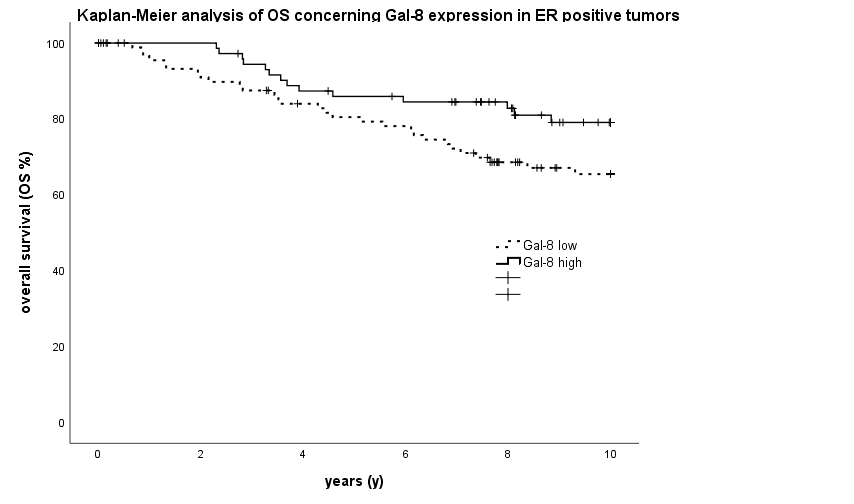


p = 0.055

**Figure S7**: Association of Gal-8 expression in the cytoplasm in ER positive tumors to the clinical outcome.
Kaplan-Meier analysis of OS in Gal-8 high and low expressing ER positive tumors (in the cytoplasm) is shown. ER positive tumors with high Gal-8 expression in the cytoplasm showed the trend to an improved OS.


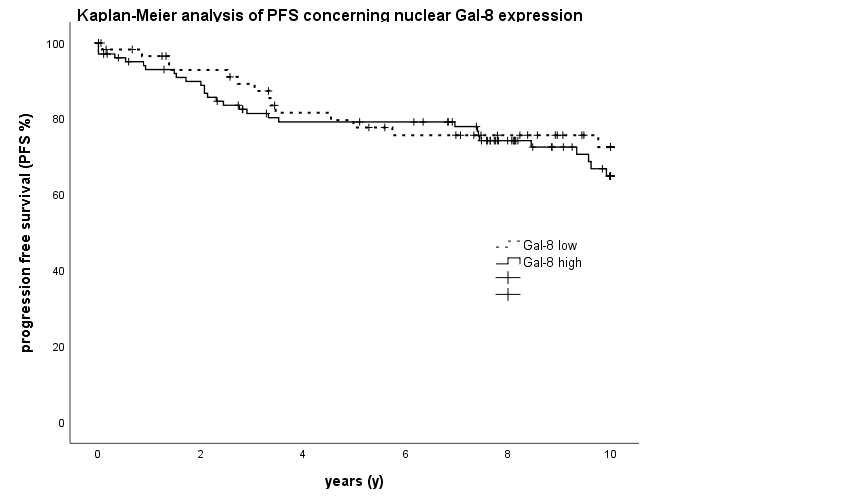


p = 0.474


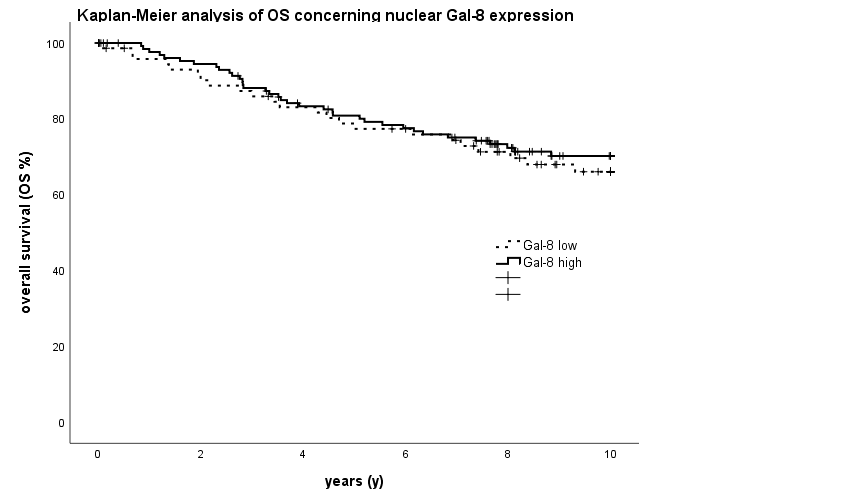


p = 0.595


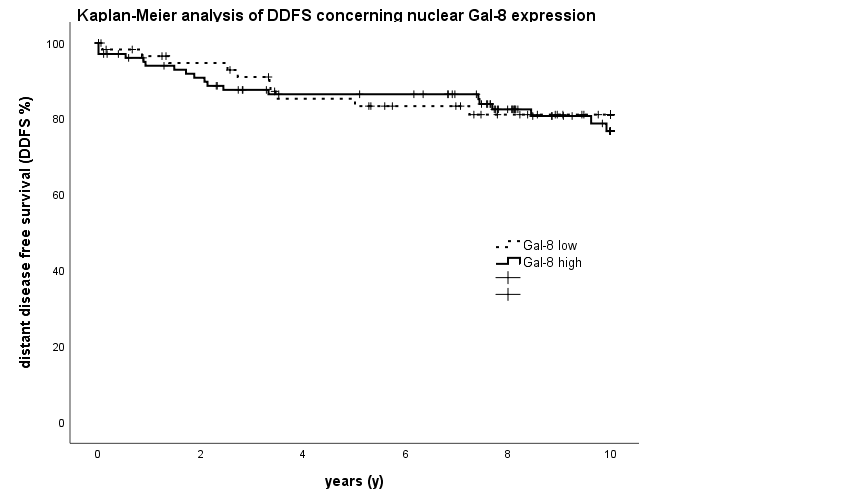


p = 0.740

**Figure S8**: Association of Gal-8 expression in the nucleus to the clinical outcome.
Kaplan-Meier analysis of OS (top left), PFS (top right) and DDFS (bottom left) in Gal-8 high and low expressing tumors (in the nucleus) is shown. No significant differences are seen.


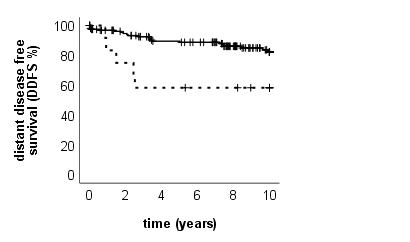

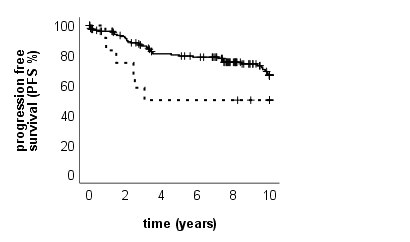

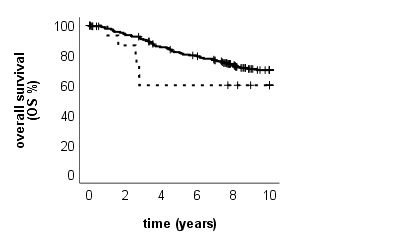

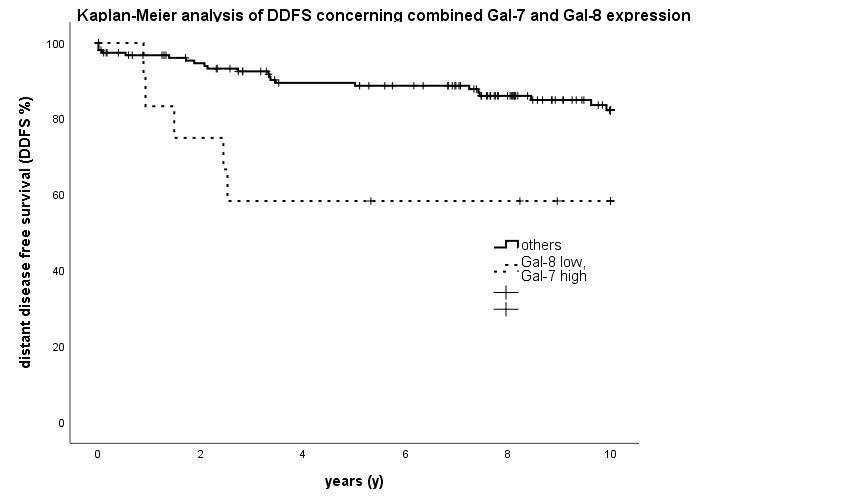


**p = 0.201**

**p = 0.067**

**p = 0.009**

**Figure S9:** OS, PFS and DDFS comparing high Gal 7 and low Gal 8 expressing tumors to the rest of the patients.
Kaplan-Meier analysis of OS (top left), PFS (top right) and DDFS (bottom left) in tumors with combined high Gal-7 expression with low Gal-8 expression (in the cytoplasm) are shown. Tumors with high Gal-7 and low Gal-8 expression in the cytoplasm show the trend of an impaired OS, PFS and a significantly reduced DDFS.


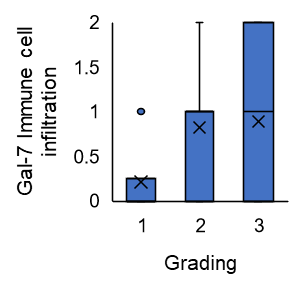

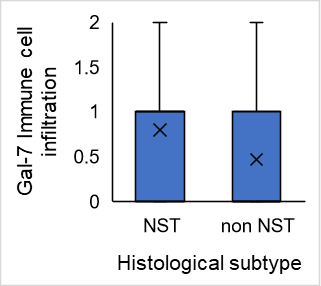

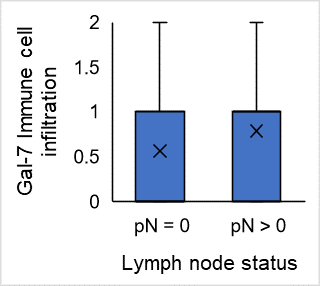


**p = 0.038**

**p = 0.001**

**p = 0.008**

A

C

B

**Figure S10**: Association of Gal 7 expression in immune cells with tumor grading, histological subtype and lymph node status.
Boxplots of the median IRS of Gal-7 staining in immune cells dependent on tumor grading (A), histological subtype (B) and lymph node status (C) are shown. Immune cells in G2/3 grading show significantly more frequent Gal-7 expression compared to G1 tumors. Gal 7 expressing immune cells were also found significantly more often in NST tumors compared to non-NST tumors and more frequently in lymph node positive tumors compared to pN0.title
